# Associations between single-nucleotide polymorphisms of human exonuclease 1 and the risk of hepatocellular carcinoma

**DOI:** 10.18632/oncotarget.13517

**Published:** 2016-11-23

**Authors:** Shengkui Tan, Ruoyun Qin, Xiaonian Zhu, Chao Tan, Jiale Song, Linyuan Qin, Liu Liu, Xiong Huang, Anhua Li, Xiaoqiang Qiu

**Affiliations:** ^1^ Department of Epidemiology, School of Public Health, Guilin Medical University, Guilin 541004, Guangxi, People's Republic of China; ^2^ Department of Epidemiology, School of Public Health, Guangxi Medical University, Nanning 530021, Guangxi, People's Republic of China; ^3^ Guangxi Center for Disease Prevention and Control, Nanning 530021, Guangxi, People's Republic of China

**Keywords:** hepatocellular carcinoma (HCC), human exonuclease 1 (*hEXO1*), single-nucleotide polymorphisms (SNPs), interaction

## Abstract

Human exonuclease 1 (*hEXO1*) is an important nuclease involved in mismatch repair system that contributes to maintain genomic stability and modulate DNA recombination. This study is aimed to explore the associations between single-nucleotide polymorphisms (SNPs) of *hEXO1* and the hereditary susceptibility of hepatocellular carcinoma (HCC). SNPs rs1047840, rs1776148, rs3754093, rs4149867, rs4149963, and rs1776181 of *hEXO1* were examined from a hospital-based case-control study including 1,196 cases (HCC patients) and 1,199 controls (non-HCC patients) in Guangxi, China. We found the rs3754093 AG genotype decreased the risk of HCC (OR=0.714, 95% CI: 0.539∼0.946). According to the results of stratification analysis, rs3754093 mutant genotype AG/GG decreased the risk of HCC with some HCC protective factors such as non-smoking, non-alcohol consumption and non-HCC family history, but also decreased the risk of HCC with HBV infection. Moreover, it was correlated to non-tumor metastasis and increased the survival of HCC patients. The results from gene-environment interaction assay indicated all *hEXO1* SNPs interacted with smoking, alcohol consumption, HBV infection in pathogenesis of HCC. However, gene-gene interaction assay suggested the interaction between rs3754093 and other 5 SNPs were associated with reducing the HCC risk. These results suggest rs3754093 exhibits a protective activity to decrease the incidence risk of HCC in Guangxi, China. In addition, all SNPs in this study interacted with environment risk factors in pathogenesis of HCC.

## INTRODUCTION

Hepatocellular carcinoma (HCC) is a very common digestive system carcinoma which is the fifth most prevalent cancer and the third most frequent cause of cancer mortality globally [1]. Each year there are approximately 630,000 new cases of HCC in the world and more than half of the new cases occur in China [2]. It is noticed that the Southern Guangxi has one of the highest occurrence of liver cancer in China [3]. Epidemiological studies suggest that the etiology of HCC is a complicated disease caused by a multi-stage process involving multiple genetic or environmental factors including alcohol consumption, tobacco use, hepatitis B virus (HBV) and hepatitis C virus (HCV) infection, as well as HCC-family history [4–7]. However, only a minority of people with established risk factors eventually develop HCC, suggesting that other environmental and/or genetic factors may play a role in HCC development. In genomic levels, dysfunctions of some oncogenes and tumor suppressor genes induced the progressive growth of malignant cells, and caused HCC.

DNA mismatch repair (MMR) is a component of the DNA repair mechanism, which plays a key role in maintaining genomic stability, preventing gene mutation, the process of DNA replication, and modulates DNA recombination and mediates cell cycle arrest [8]. Human exonuclease 1 (*hEXO1*) which located at chromosome 1q42-q43, contains one untranslated exon followed by 13 coding exons and encodes an 846 amino acid protein [9–11]. *EXO1* is an important member of DNA MMR system which has been well documented to implicate in DNA replication, DNA repair, DNA restructuring, and maintain the stability of telomeres [12]. *EXO1* can interact physically with the MMR proteins MSH2 and MLH1 in yeast and human cells, and with MSH3 in human cells [9, 13–17]. Wei indicated that mammalian *EXO1* is responsible for mutation prevention and mice *EXO1* inactivation reduced survival time and increased risk of lymphoma [18]. In addition, some studies have indicated SNPs of *hEXO1* were associated with various cancers including lung cancer, colorectal cancer, gastric cancer, breast cancer, oral cancer, and other tumors [10, 19–25]. A number of studies have reported a SNP of the *hEXO1* gene, K589E (rs1047840), is associated with human lung cancer, breast cancer, oral cancer and gastric cancer risk in Chinese Taiwan population [24, 26–28]. Jin [29] and Luo [30] reported the *hEXO1* K589E was associated with human lung cancer and cervical cancer susceptibility in Chinese Mainland population. The *hEXO1* K589E polymorphism may be a genetic susceptibility factor of HCC in the Turkish population [31], and increase the risk of colorectal cancer in the Polish population [32].

However, the association between SNPs of *hEXO1* and hereditary susceptibility of HCC has not been investigated in China. In this study, we conducted a screening on *hEXO1* from NIEHS database to seek candidate SNPs in Chinese population. Minor allele frequency (MAF) of six SNPs (rs1047840, rs1776148, rs3754093, rs4149867, rs4149963, and rs1776181) was greater than 0.05 in Chinese population and had potential functions. Therefore, these 6 SNPs were selected to investigate their frequency distributions and associations with HCC in Guangxi, China. We expect the study will provide scientific basis for prevention and treatment of HCC.

## RESULTS

### Demographic information of HCC patients and controls

The demographic information of this study is presented in Table 1. There were no statistical differences in the age, sex and nation distribution between HCC patients and controls (*P*>0.05). However, 4 risk factors including HBV infection, alcohol consumption, smoking and HCC family history showed a significantly statistical differences between HCC patients and controls (*P*<0.05).

**Table 1:** Demographic information of the study objects

Characteristics	Controls (n=1199)	Cases (n=1196)	*t/χ*^2^	*P*
Age(years, $\bar{X}$ ± s)	48.28±11.69	48.58±10.84	-0.648	0.517
Gender			0.042	0.837
Male	1045	1039		
Female	154	157		
Nation			3.005	0.223
Han	753	791		
Zhuang	423	386		
Others	23	19		
Smoking			140.90	**0.000**
Yes	1008	749		
No	191	447		
Alcohol Consumption			141.74	**0.000**
Yes	1036	785		
No	163	411		
HBV infection			1375.31	**0.000**
Yes	98	195		
No	1101	1001		
HCC family history			42.76	**0.000**
Yes	1189	1130		
No	10	66		

### Distribution of genotypes and risk of HCC

The allele and genotype distributions of the six SNPs in this study and their associations with HCC risk are presented at Table 2. The distribution of all the SNPs’ genotypes in the controls obeyed Hardy-Weinberg equilibrium (HWE) (*P*> 0.05). The chi-square test also showed distribution of all the SNPs’ genotypes in the cases and the controls had no statistic difference (*P*>0.05). Following adjust the nation, gender, age, HBV infection, alcohol consumption, smoking, and HCC family history in Logistic regression models, the rs3754093AG (adjusted OR=0.714; 95% CI=0.539-0.946) genotypes exhibited an activity to reduce the risk of HCC (*P*<0.05).

**Table 2:** Distribution of genotypes and risk of HCC

Genotypes	Controls, n (%)	Cases, n (%)	OR (95%CI)^a^	OR (95%CI)^b^
rs1047840				
AA	54(4.50)	64(5.35)	1.000	1.000
AG	378(31.53)	388(32.44)	0.866(0.587∼1.278)	0.769(0.419∼1.410)
GG	767(63.97)	744(62.21)	0.818(0.562∼1.192)	0.789(0.440∼1.417)
AG/GG	1145(95.50)	1132(94.65)	0.834(0.575∼1.209)	0.782(0.439∼1.394)
rs1776148				
AA	38(3.17)	43(3.60)	1.000	1.000
AG	362(30.19)	355(29.68)	0.867(0.547∼1.373)	0.614(0.312∼1.207)
GG	799(66.64)	798(66.72)	0.883(0.564∼1.380)	0.547(0.284∼1.054)
AG/GG	1161(96.83)	1153(96.40)	0.878(0.563∼1.368)	0.568(0.297∼1.086)
rs3754093				
AA	401(33.44)	441(36.87)	1.000	1.000
AG	580(48.37)	557 (46.57)	0.873(0.731∼1.044)	**0.714(0.539∼0.946)**
GG	218(18.18)	198(16.55)	0.826(0.653∼1.045)	0.822(0.567∼1.193)
AG/GG	798(66.56)	755(63.12)	0.906(0.766∼1.071)	**0.741 (0.569∼0.966)**
rs4149867				
CC	858(71.56)	718(68.39)	1.000	1.000
CT	316(26.35)	347 (29.01)	1.312(1.094∼1.574)	0.962 (0.723∼1.281)
TT	25(2.09)	31(2.59)	1.482(0.867∼2.533)	0.767(0.337∼1.749)
CT/TT	341(28.44)	378(31.60)	1.325(1.110∼1.581)	0.945 (0.715∼1.248)
rs4149963				
CC	1057(88.15)	1047(87.54)	1.000	1.000
CT	129(10.76)	124(10.37)	0.970(0.748∼1.260)	0.805 (0.531∼1.222)
TT	13(1.09)	25(2.09)	1.941(0.988∼3.815)	1.270(0.410∼3.932)
CT/TT	142(11.85)	149(12.46)	1.059(0.829∼1.354)	0.846 (0.570∼1.257)
rs1776181				
CC	644(53.71)	609(50.92)	1.000	1.000
CT	445(37.11)	447(37.37)	1.062(0.895∼1.261)	0.939 (0.716∼1.230)
TT	110(9.17)	140(11.71)	1.346(1.024∼1.769)	0.873(0.563∼1.353)
CT/TT	555(46.28)	587(49.08)	1.118(0.953∼1.313)	0.925 (0.717∼1.192)

^a^:OR and 95%CI without adjusting. ^b^:Adjusted by logistic regression for age, gender, nations, smoking, alcohol consumption, HBV infection, and HCC family history.

### Stratification analysis

The distribution of HCC risk factors including smoking, alcohol consumption, HBV infection, and HCC family history in the cases and the controls had statistic difference. Therefore, we conducted a stratification analysis based on these factors (Table 3 and Supplementary Tables S1-S5). As shown in Table 3, the interaction of rs3754093 and HCC risk factors was assessed for the possible combined effect on HCC risk.

**Table 3:** Results of rs3754093 stratification analysis

Variables	Genotypes	Controls (n=1199)	Cases (n=1196)	OR (95%CI)^a^	*P*
Smoking					
No	AA	336	278	1.000	**0.029**
Yes	AA	65	163	1.000	0.246
Alcohol Consumption					
No	AA	345	296	1.000	**0.005**
Yes	AA	56	145	1.000	0.716
HBV Infection					
No	AA	377	73	1.000	0.417
Yes	AA	24	368	1.000	**0.010**
HCC family history					
No	AA	399	413	1.000	**0.039**
Yes	AA	2	28	1.000	0.544

^a^: Adjusted by logistic regression for age, gender, nations, smoking, alcohol consumption, HBV infection, and HCC family history.

### Associations between *hEXO1* SNPs and the clinicopathological features of HCC

**Figure 1:**
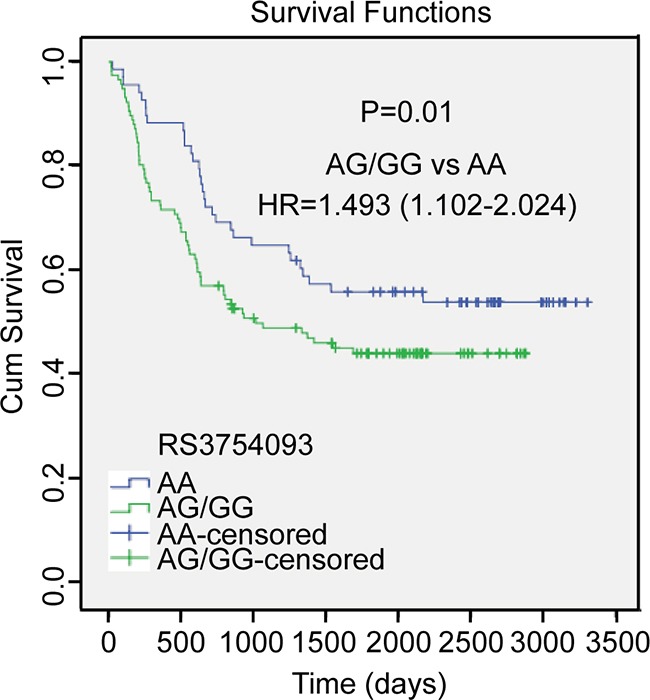
Kaplan-Meier overall survival curve for HCC patients based on rs3754093 genotypes *P* value is from the log-rank test. HR with 95% CI was from univariate analysis of OS and DFS.

We evaluated the associations of *hEXO1* SNPs with various clinicopathological features of HCC including: tumor size, tumor number, TNM staging (tumor-node-metastasis staging, a cancer staging notation system that describes the stage of a cancer which originates from a solid tumor with alphanumeric codes), combined with liver cirrhosis, AFP level, and tumor metastasis (Table 4∼9). The G allele (AG/GG genotype) of rs3754093 was associated with non-tumor metastasis (adjusted OR: 1.535, 95% CI: 1.023–2.538, *P*<0.05). However, other 5 SNPs of *hEXO1* were not significantly associated with any of the clinicopathological features. Because tumor metastasis is directly related to the prognosis of HCC patients, we performed a univariate survival analysis shown in Figure 1. In accordance with above results, HCC patients with allele G (AG/GG genotype) had a significantly increased risk for death than patients without allele G (AA genotype) (HR= 1.493, 95% CI = 1.102–2.024, P=0.01) (Figure 1).

**Table 4:** The associations between rs3754093 and clinical characteristics of hepatocellular carcinoma patients

Variables	AA	AG/GG	OR(95%CI)^a^	OR(95%CI)^b^
tumor size				
≥5cm	85(34.56)	161(63.44)	1.000	1.000
<5cm	217(29.17)	527(70.83)	1.282(0.944∼1.742)	1.165 (0.876∼1.963)
tumor number				
solitary	267(30.58)	606(69.42)	1.000	1.000
multiple	35(29.91)	82(70.09)	1.032(0.677∼1.573)	0.972 (0.596∼2.248)
TNM staging				
T1+T2	262(31.30)	575(68.70)	1.000	1.000
T3+T4	40(26.14)	113(74.86)	1.287(0.872∼1.899)	1.196 (0.756∼1.972)
Combined with liver cirrhosis				
Yes	100(23.98)	317(76.02)	1.000	1.000
No	202(35.25)	371(64.75)	**0.579(0.437∼0.769)**	0.585 (0.446∼0.756)
AFP level (ng/ml)				
≥400	205 (29.45)	491(70.55)	1.000	1.000
<400	97 (37.16)	164(62.84)	**0.706 (0.523∼0.952)**	0.681 (0.434∼1.051)
tumor metastasis				
Yes	53(40.15)	79(59.85)	1.000	1.000
No	249(29.02)	609(70.98)	**1.641(1.124∼2.394)**	**1.535 (1.023∼2.538)**

^a^: OR (95% CI) not adjusted; ^b^: OR (95% CI) adjusted for age, gender, nations, smoking, alcohol consumption, HBV infection, and HCC family history.

**Table 5:** The associations between rs1047840 and clinical characteristics of hepatocellular carcinoma patients

Variables	AA	AG/GG	OR(95%CI)^a^	OR(95%CI)^b^
tumor size				
≥5cm	18(7.32)	228(92.68)	1.000	1.000
<5cm	33(4.64)	711(95.36)	0.588(0.325∼1.064)	0.582(0.202∼1.678)
tumor number				
solitary	48(5.50)	825(94.50)	1.000	1.000
multiple	3(2.56)	114(97.44)	2.211(0.678∼7.215)	2.131(0.268∼16.913)
TNM staging				
T1+T2	48(5.73)	789(94.27)	1.000	1.000
T3+T4	3(1.96)	150(98.04)	3.042(0.935∼9.893)	2.828(0.359∼22.287)
Combined with liver cirrhosis				
Yes	21(5.04)	396(94.36)	1.000	1.000
No	30(5.24)	543(94.86)	0.960(0.541∼1.702)	1.115(0.402∼3.089)
AFP level (ng/ml)				
≥400	33(4.74)	663(95.26)	1.000	1.000
<400	18(6.89)	243(93.10)	0.672(0.371∼1.216)	0.749(0.264∼2.130)
tumor metastasis				
Yes	6(4.55)	126(95.45)	1.000	1.000
No	45(5.24)	813(94.76)	1.162(0.486∼2.781)	1.201(0.257∼5.681)

^a^: OR (95% CI) not adjusted; ^b^: OR (95% CI) adjusted for age, gender, nations, smoking, alcohol consumption, HBV infection, and HCC family history.

**Table 6:** The associations between rs1776148 and clinical characteristics of hepatocellular carcinoma patients

Variables	AA	AG/GG	OR(95%CI)^a^	OR(95%CI)^b^
tumor size				
≥5cm	6(2.44)	240(97.56)	1.000	1.000
<5cm	24(3.23)	720(96.77)	1.333(0.539∼3.301)	1.587(0.317∼7.937)
tumor number				
solitary	27(3.09)	846(96.91)	1.000	1.000
multiple	3(2.56)	114(97.44)	1.213(0.362∼4.062)	1.028(0.121∼8.760)
TNM staging				
T1+T2	30(3.58)	807(96.42)	1.000	1.000
T3+T4	6(3.92)	147(96.08)	0.911(0.373∼2.227)	1.128(0.569∼2.233)
Combined with liver cirrhosis				
Yes	3(0.72)	414(99.28)	1.000	1.000
No	27(4.71)	546(95.29)	**0.147(0.044∼0.486)**	0.154(0.019∼1.260)
AFP level (ng/ml)				
≥400	27(3.88)	669(96.12)	1.000	1.000
<400	3(1.02)	291(98.98)	**3.915(1.178∼13.007)**	4.391(0.536∼35.971)
tumor metastasis				
Yes	6(4.55)	126(95.45)	1.000	1.000
No	30(3.50)	828(96.50)	0.761(0.310∼1.865)	0.764(0.164∼3.606)

^a^: OR (95% CI) not adjusted; ^b^: OR (95% CI) adjusted for age, gender, nations, smoking, alcohol consumption, HBV infection, and HCC family history.

**Table 7:** The associations between rs4149867 and clinical characteristics of hepatocellular carcinoma patients

Variables	AA	AG/GG	OR(95%CI)^a^	OR(95%CI)^b^
tumor size				
≥5cm	165(67.07)	81(32.93)	1.000	1.000
<5cm	501(67.34)	243(32.66)	1.125(0.533∼2.373)	0.638(0.357∼1.138)
tumor number				
solitary	609(69.76)	264(30.24)	1.000	1.000
multiple	78(66.66)	39(33.34)	1.153(0.765∼1.739)	1.080(0.521∼2.238)
TNM staging				
T1+T2	588(70.25)	249(29.75)	1.000	1.000
T3+T4	96(64.00)	54(36.00)	1.328(0.922∼1.913)	1.250(0.646∼2.419)
Combined with liver cirrhosis				
Yes	273(65.94)	141(34.06)	1.000	1.000
No	414(72.25)	159(27.05)	**0.744(0.566∼0.977)**	0.781(0.480∼1.270)
AFP level (ng/ml)				
≥400	483(69.40)	213(30.60)	1.000	1.000
<400	204(69.39)	90(30.61)	1.000(0.744∼1.345)	0.988(0.587∼1.665)
tumor metastasis				
Yes	99(75.00)	33(25.00)	1.000	1.000
No	582(68.31)	270(31.69)	0.719(0.472∼1.093)	0.726(0.348∼1.515)

^a^: OR (95% CI) not adjusted; ^b^: OR (95% CI) adjusted for age, gender, nations, smoking, alcohol consumption, HBV infection, and HCC family history.

**Table 8:** The associations between rs4149963 and clinical characteristics of hepatocellular carcinoma patients

Variables	AA	AG/GG	OR(95%CI)^a^	OR(95%CI)^b^
tumor size				
≥5cm	218(88.62)	28(11.38)	1.000	1.000
<5cm	669(89.91)	75(10.08)	1.146(0.723∼1.815)	1.648(0.775∼3.508)
tumor number				
solitary	780(89.34)	93(10.65)	1.000	1.000
multiple	104(88.89)	13(11.11)	1.048(0.567∼1.940)	1.610(0.614∼4.220)
TNM staging				
T1+T2	738(88.17)	99(11.83)	1.000	1.000
T3+T4	131(87.33)	19(2.67)	1.081(0.640∼1.828)	0.586(0.189∼1.814)
Combined with liver cirrhosis				
Yes	360(86.96)	51(13.04)	1.000	1.000
No	516(90.05)	57(9.95)	0.780(0.522∼1.164)	0.759(0.378∼1.527)
AFP level (ng/ml)				
≥400	603(86.64)	93(13.36)	1.000	1.000
<400	252(85.71)	42(14.29)	1.081(0.730∼1.601)	0.420(0.168∼1.049)
tumor metastasis				
Yes	117(88.64)	15(11.36)	1.000	1.000
No	762(88.81)	96(11.19)	1.018(0.571∼1.814)	1.308(0.506∼3.382)

^a^: OR (95% CI) not adjusted; ^b^: OR (95% CI) adjusted for age, gender, nations, smoking, alcohol consumption, HBV infection, and HCC family history.

**Table 9:** The associations between rs1776181 and clinical characteristics of hepatocellular carcinoma patients

Variables	AA	AG/GG	OR(95%CI)^a^	OR(95%CI)^b^
tumor size				
≥5cm	390(52.42)	354(41.58)	1.000	1.000
<5cm	120(48.78)	126(51.22)	1.157(0.867∼1.544)	1.183 (0.711∼1.966)
tumor number				
solitary	450(51.55)	423(48.45)	1.000	1.000
multiple	60(51.28)	57(48.72)	1.101(0.687∼1.487)	0.919 (0.464∼1.819)
TNM staging				
T1+T2	427(49.82)	410(50.18)	1.000	1.000
T3+T4	80(60.00)	70(40.0)	0.911(0.643∼1.291)	0.850 (0.545∼1.426)
Combined with liver cirrhosis				
Yes	231(55.80)	183(44.20)	1.000	1.000
No	279(48.69)	294(51.31)	**1.330(1.032∼1.715)**	1.295 (0.828∼2.028)
AFP level (ng/ml)				
≥400	357(51.29)	339(48.71)	1.000	1.000
<400	153(52.04)	141(47.96)	0.971(0.739∼1.275)	0.882 (0.597∼1.558)
tumor metastasis				
Yes	72(54.55)	60(45.45)	1.000	1.000
No	435(51.06)	423(48.94)	0.857(0.593∼1.239)	0.857 (0.450∼1.632)

^a^: OR (95% CI) not adjusted; ^b^: OR (95% CI) adjusted for age, gender, nations, smoking, alcohol consumption, HBV infection, and HCC family history

### Gene-environment interaction and SNP-SNP interaction

As shown in Table 10, all the SNPs were found to interact with smoking, alcohol consumption, HBV infection in the pathogenesis of HCC. The results of SNP-SNP interaction suggested the interaction between rs3754093 and other 5 SNPs (rs1047840, rs1776148, rs4149867, rs4149963 and rs1776181) was associated with reducing the HCC risk (Table 11).

**Table 10:** Gene-environment interaction

Factors	*β*	*S.E*.	*Wald χ^2^*	OR (95%CI) ^a^	*P*
rs1047840 × Smoking	0.428	0.093	21.020	1.534(1.278∼1.842)	**0.000**
rs1047840 × Alcohol Consumption	0.336	0.096	12.225	1.399(1.159∼1.689)	**0.000**
rs1047840 × HBV Infection	2.102	0.074	809.417	8.818(7.078∼9.456)	**0.000**
rs1047840 × HCC family history	0.229	0.235	0.949	1.257(0.793∼1.293)	0.330
rs1776148 × Smoking	0.424	0.093	20.652	1.527(1.272∼1.833)	**0.000**
rs1776148 × Alcohol Consumption	0.318	0.095	11.066	1.374(1.139∼1.657)	**0.001**
rs1776148 × HBV Infection	2.080	0.073	820.029	8.003(6.941∼9.288)	**0.000**
rs1776148 × HCC family history	0.236	0.223	1.022	1.266(0.801∼2.001)	0.312
rs3754093 × Smoking	0.421	0.103	16.756	1.524(1.245∼1.864)	**0.000**
rs3754093 × Alcohol Consumption	0.367	0.105	12.101	1.443(1.174∼1.775)	**0.001**
rs3754093 × HBV Infection	2.326	0.092	641.686	10.235(8.549∼12.253)	**0.000**
rs3754093 × HCC family history	0.186	0.269	0.477	1.204(0.711∼2.041)	0.490
rs4149867 × Smoking	0.462	0.124	13.852	1.587(1.244∼2.023)	**0.000**
rs4149867 × Alcohol Consumption	0.385	0.134	8.240	1.469(1.130∼1.910)	**0.004**
rs4149867 × HBV Infection	3.238	0.126	664.917	25.485(19.925∼32.597)	**0.000**
rs4149867 × HCC family history	0.391	0.340	1.324	1.478(0.760∼2.876)	0.250
rs4149963 × Smoking	0.610	0.149	16.769	1.840(1.374∼2.464)	**0.000**
rs4149963 × Alcohol Consumption	0.454	0.159	8.276	1.580(1.157∼2.158)	**0.004**
rs4149963 × HBV Infection	3.707	0.134	762.612	40.703(31.296∼52.965)	**0.000**
rs4149963 × HCC family history	0.350	0.401	0.760	1.419(0.646∼3.113)	0.383
rs1776181 × Smoking	0.497	0.110	20.283	1.644(1.324∼2.042)	**0.000**
rs1776181 × Alcohol Consumption	0.347	0.115	9.139	1.414(1.130∼1.771)	**0.003**
rs1776181 × HBV Infection	2.578	0.103	620.820	13.175(10.756∼16.137)	**0.000**
rs1776181 × HCC family history	0.372	0.316	1.384	1.450(0.781∼2.695)	0.239

^a^: Adjusted by logistic regression for age, gender, nations, smoking, alcohol consumption, HBV infection, and HCC family history.

**Table 11:** SNP-SNP interaction on HCC risk

Factors	*β*	*S.E*.	*Wald χ^2^*	OR (95%CI) ^a^	*P*
rs1047840 × rs1776148	-0.202	0.113	3.190	0.817(0.654∼1.020)	0.074
rs1047840 × rs3754093	-0.148	0.065	5.262	0.862(0.759∼0.979)	**0.022**
rs1047840 × rs4149867	-0.055	0.069	0.627	0.947(0.826∼1.084)	0.429
rs1047840 × rs4149963	-0.098	0.096	1.045	0.906(0.751∼1.094)	0.307
rs1047840 × rs1776181	-0.061	0.063	0.924	0.941(0.831∼1.065)	0.337
rs1776148 × rs3754093	-0.177	0.065	7.302	0.838(0.737∼0.953)	**0.007**
rs1776148 × rs4149867	-0.052	0.070	0.547	0.950(0.828∼1.089)	0.460
rs1776148 × rs4149963	-0.151	0.098	2.367	0.860(0.709∼1.042)	0.124
rs1776148 × rs1776181	-0.067	0.063	1.104	0.937(0.827∼1.059)	0.293
rs3754093 × rs4149867	-0.134	0.065	4.193	0.875(0.769∼0.994)	**0.041**
rs3754093 × rs4149963	-0.171	0.084	4.153	0.843(0.715∼0.993)	**0.042**
rs3754093 × rs1776181	-0.128	0.059	4.660	0.880(0.783∼0.988)	**0.031**
rs4149867 × rs4149963	-0.104	0.098	1.129	0.901(0.744∼1.092)	0.288
rs4149867 × rs1776181	-0.090	0.060	2.301	0.914(0.813∼1.027)	0.129
rs4149963 × rs1776181	-0.066	0.083	2.628	0.936(0.795∼1.102)	0.428

^a^: Adjusted by logistic regression for age, gender, nations, smoking, alcohol consumption, HBV infection, and HCC family history.

## DISCUSSION

In this study, we investigated six SNPs of the *hEXO1* gene and their association with hereditary susceptibility for HCC in the population of Guangxi, China. Among these six SNPs, we found rs3754093 was significantly associated with a higher susceptibility, and exhibited a protective activity in HCC patients.

*hEXO1* is an endonuclease which plays a critical role in both 5′-3′ and 3′-5′ mispair-dependent excision repair to maintain the overall integrity of the MMR protein complex in human MMR system [33, 34]. Alteration of *MMR* gene can cause cell DNA mismatch repair by increasing cell spontaneous mutation frequency, down-regulating tumor suppressor genes and up-regulating oncogenes, eventually leading to the occurrence of tumors [35]. Therefore, *hEXO1* gene is an important target gene in association with the risk of various malignancies [23, 25, 36]. Moreover, *hEXO1* activates mutations of target genes through cutting DNA of telomere [43], which is a kind of special protein-DNA structure that exists in eukaryotic cell linear chromosomes [37] and plays an important role in maintaining cell proliferation ability and protecting chromosome integrity [38]. Through its role in these recombinational events, such as repairing of DNA double-strand breaks and maintaining of telomere stabilization, *hEXO1* functions in carcinogenesis of various tumors [18, 26].

In consistent with its key role in carcinogenesis, *hEXO1* rs3754093 showed a protective activity and decreased the risk for death in HCC patients in our study. Furthermore, function prediction showed *hEXO1* rs3754093 was a transcription factor binding site (TFBS). Previous reports showed that SNPs on TFBS could alter the affinity between transcription factors and specific DNA sequences, then cause expression alterations of specific genes [39–41]. That is to say, the polymorphism of rs3754093 could alter the expression level of *hEXO1* through changing its binding affinity to transcription factors. And then the expression change of *hEXO1* might either alter the procedure of MMR to result in gene mutations [42], or increase the risk of cancer through telomere cutting to cause genomic instability [13]. This might be the mechanism of association between *hEXO1* rs3754093 and HCC. In addition, we did not find the other 5 SNPs associated with HCC pathogenesis, indicating that the 5 SNPs didn't alter expression level of *hEXO1*.

From gene-environment interaction analysis, we found the six *hEXO1* SNPs were associated with such environment factors, smoking, alcohol consumption and HBV infection. These environmental factors are well documented risk factors of HCC and each of them is an independent strong cause of HCC as previous studies showed. Meta-analysis of epidemiologic studies suggested tobacco use was associated with the increasing of HCC risk [43]. Koh also reported that the tobacco use increased the risk of HCC in the Singapore Chinese population [44]. Both alcohol and tobacco are synergistic risk factors for HCC [45]. In addition, the HBV and HCV infection also plays an important role in increasing the risk of HCC [46], and also is a major factor of HCC risk in Guangxi, China [47]. The chronic HBV infection resulted in the hepatic inflammation reaction, fibrosis, hepatocirrhosis and finally induced liver cancer [46]. We draw a similar conclusion on the effects of these environment factors in development of HCC with previous reports. Moreover, all the six *hEXO1* SNPs in the study have interaction effects with some environment factors on HCC risk.

Previous studies have reported the SNP-SNP interaction was significantly associated with the pathological process of breast cancer [48], colon cancer [49], oesophageal squamous cell carcinoma [50] and HCC [51], respectively. We found the interaction between rs3754093 and other SNPs (rs1047840, rs1776148, rs4149867, rs4149963 and rs1776181) was significantly associated with reduced the risk of HCC. These results suggested the SNP-SNP interaction may decrease the HCC risk.

HCC is a serious cancer with a complicated pathogenesis related to lots of genes and environmental factors. However, the mechanism of HCC incidence has not been totally understood. In this study, we found the *hEXO1* rs3754093 was a protective factor to reduce the risk of HCC. Moreover, the combined effects of SNP-SNP interactions may decrease the risk of HCC in Guangxi, which has a high incidence of HCC in China. As the association between rs3754093 and the risk of HCC is only an epidemiological link right now, further function study on the SNP should be conducted to validate the current finding.

## MATERIALS AND METHODS

### Study subjects

The cases (n=1,199) were newly diagnosed as HCC patients without chemotherapy and radiotherapy who recruited from the Affiliated Tumor Hospital of Guangxi Medical University between June 2007 and March 2014. All HCC patients were diagnosed according to the World Health Organization (WHO) Classification of HCC diagnostic criteria. The controls (n=1,196) were non-tumor patients from the First Affiliated Hospital of Guangxi Medical University during the same period. Inclusion and exclusion criteria for participants: 1. Inclusion criteria: (1) Cases: new HCC patients hospitalized in the period of study, and were pathologically confirmed. (2) Controls: patients without HCC hospitalized in the same period with the cases. 2. Exclusion criteria: (1) Cases: have been treated, HCV infected, with other tumors. (2) Controls: suffering from cancer, HCV infected. This study was respectively approved by the Institutional Review Board of the Affiliated Tumor Hospital of Guangxi Medical University and First Affiliated Hospital of Guangxi Medical University. The informed consent was obtained from each subject in this study.

### Questionnaire survey and blood sample collection

Questionnaires were designed after consulting experts. Data of questionnaire survey was collected from a structured interview, and conducted by trained interviewers after a pre-investigation, which including the demographic characteristics (name, nation, gender, age), smoking alcohol consumption, hepatitis B virus (HBV) infection and HCC family history. 2 mL peripheral blood sample was collected from each study object. Genome DNA was extracted from the blood samples. All laboratory and questionnaire data were coded, entered by two investigators with a logical and consistency test, and verified using EpiData 3.1 (www.epidata.dk/download.php). Neither the laboratory nor the data entry personnel had any knowledge of the subjects’ case-control status.

### DNA extraction and genotyping

The blood was collected from the all subjects using an EDTA-K_2_ anticoagulant blood vessel. The DNA was extracted according to the phenol-chloroform method and stored at -80°C. The genotyping was performed in an Applied Biosystems 7500 Fast Real-Time quantitative polymerase chain reaction (qPCR) system with the following TaqMan SNP Genotyping Assays kit (Applied Biosystems) according to the manufacturer's instructions. Each PCR reaction mixture (10 μL) contained DNA template (0.4 μL), ddH_2_O (4.35 μL), 2×TaqMan Universal PCR Master Mix (5 μL), and 20× SNP Genotyping Assay Mix (0.25 μL). The genomic DNA was amplified at 95°C for 10 min, followed by 40 cycles of 95°C for 15s and 60°C for 60s. All genotyping reagents and analytical software were purchased from Applied Biosystems. Approximately 5% of the samples were randomly repeated to validate the genotyping procedures, and the concordance rate was 100%. The results of the genotyping were analyzed with 7500 Fast System V1.4.0 SDS software.

### Survival analysis

356 HCC patients were brought into a survival analysis cohort. The patients were followed up from July 2007 to May 2016. Survival time was counted the first day after surgery and end up when the patients appeared metastasis and recurrence or death. Survival time was calculated in months. Till the end of follow-up, a total of 31 patients were lost to follow-up, 325 patients with complete follow-up data, follow-up rate was 91.3%.

### Statistical analysis

Data entry and consistency check were conducted on the EpiData3.1 software. The SPSS 20.0 software was used for the statistical analyses. Logistic regression models were used to estimate odds ratio (OR) and 95% confidence interval (CI). Gene-environment interactions and gene-gene interactions were performed through multiplicative model of binary logistic regression. Hardy-Weinberg Equilibrium (HWE) in the controls was tested by Haploview 4.2 software. Kaplan–Meier method, Log–rank test and COX regression were used on survival analysis.

## SUPPLEMENTARY TABLES


