# Supplementary material for: Associations between single-nucleotide polymorphisms of human exonuclease 1 and the risk of hepatocellular carcinoma
Source: Oncotarget. 2016 Nov 23;7(52):87180–93. doi: 10.18632/oncotarget.13517 (PMC5349980; doi:10.18632/oncotarget.13517)
Supplement: Supplementary file 1 [file oncotarget-07-87180-s001.pdf]

Associations between single-nucleotide polymorphisms of human exonuclease 1 and the risk of hepatocellular carcinoma

SUPPLEMENTARY TABLES

Supplementary Table S1: Results of rs1047840 stratification analysis

Variables	Genotypes	Controls (n=1199)	Cases (n=1196)	OR (95%CI)[a]	*P*
Smoking					
No	AA	46	37	1.000	0.595
	AG/GG	962	712	0.828(0.412~1.661)	
Yes	AA	8	27	1.000	0.517
	AG/GG	183	420	0.707(0.248~2.019)	
Alcohol Consumption					
No	AA	47	41	1.000	0.452
	AG/GG	989	744	0.773(0.395~1.513)	
Yes	AA	7	23	1.000	0.868
	AG/GG	156	388	0.908(0.291~2.834)	
HBV Infection					
No	AA	48	13	1.000	0.190
	AG/GG	1053	182	0.636(0.323~1.252)	
Yes	AA	6	51	1.000	0.676
	AG/GG	92	950	1.208(0.498~2.930)	
HCC family history					
No	AA	54	59	1.000	0.467
	AG/GG	1135	1071	0.805(0.449~1.443)	
Yes	AA	1	5	1.000	0.418
	AG/GG	9	61	0.951(0.843~1.073)	

[a]: Adjusted by logistic regression for age, gender, nations, smoking, alcohol consumption, HBV infection, and HCC family history.

Supplementary Table S2: Results of rs1776148 stratification analysis

Variables	Genotypes	Controls (n=1199)	Cases (n=1196)	OR (95%CI)[a]	*P*
Smoking					
No	AA	32	27	1.000	0.225
	AG/GG	976	722	0.618(0.284~1.346)	
Yes	AA	6	16	1.000	0.246
	AG/GG	185	431	0.485(0.143~1.646)	
Alcohol Consumption					
No	AA	35	29	1.000	0.357
	AG/GG	1001	756	0.302(0.331~1.489)	
Yes	AA	3	14	1.000	0.054
	AG/GG	160	397	0.224(0.049~1.024)	
HBV Infection					
No	AA	37	10	1.000	0.227
	AG/GG	1064	185	0.633(0.301~1.330)	
Yes	AA	1	33	1.000	0.237
	AG/GG	97	968	0.297(0.040~2.217)	
HCC family history					
No	AA	38	39	1.000	0.101
	AG/GG	1151	1091	0.579(0.301~1.113)	
Yes	AA	2	4	1.000	0.451
	AG/GG	8	62	0.955(0.846~1.077)	

[a]: Adjusted by logistic regression for age, gender, nations, smoking, alcohol consumption, HBV infection, and HCC family history.

Supplementary Table S3: Results of rs4149867 stratification analysis

Variables	Genotypes	Controls (n=1199)	Cases (n=1196)	OR (95%CI)[a]	*P*
Smoking					
No	CC	727	509	1	0.845
	CT/TT	281	240	1.034(0.741~1.443)	
Yes	CC	131	309	1	0.313
	CT/TT	60	138	0.768(0.461~1.282)	
Alcohol Consumption					
No	CC	47	41	1	0.894
	CT/TT	989	744	0.978(0.710~1.349)	
Yes	CC	7	23	1	0.568
	CT/TT	156	388	0.847(0.479~1.498)	
HBV Infection					
No	CC	48	13	1	0.128
	CT/TT	1053	182	0.748(0.514~1.088)	
Yes	CC	6	51	1	0.241
	CT/TT	92	950	1.321(0.829~2.102)	
HCC family history					
No	CC	54	59	1	0.637
	CT/TT	1135	1071	0.935(0.706~1.237)	
Yes	CC	1	5	1	0.598
	CT/TT	9	61	2.274(1.107~48.30)	

[a]: Adjusted by logistic regression for age, gender, nations, smoking, alcohol consumption, HBV infection, and HCC family history.

Supplementary Table S4: Results of rs4149963 stratification analysis

Variables	Genotypes	Controls (n=1199)	Cases (n=1196)	OR (95%CI)[a]	*P*
Smoking					
No	CC	893	657	1	0.620
	CT/TT	115	92	0.886(0.549~1.430)	
Yes	CC	164	390	1	0.493
	CT/TT	27	57	0.783(0.390~1.574)	
Alcohol Consumption					
No	CC	47	41	1	0.741
	CT/TT	989	744	0.926(0.587~1.462)	
Yes	CC	7	23	1	0.302
	CT/TT	156	388	0.663(0.304~1.447)	
HBV Infection					
No	CC	48	13	1	0.829
	CT/TT	1053	182	0.945(0.566~1.578)	
Yes	CC	6	51	1	0.283
	CT/TT	92	950	0.729(0.410~1.297)	
HCC family history					
No	CC	54	59	1	0.456
	CT/TT	1135	1071	0.859(0.577~1.297)	
Yes	CC	1	5	1	0.196
	CT/TT	9	61	0.135(0.007~2.804)	

[a]: Adjusted by logistic regression for age, gender, nations, smoking, alcohol consumption, HBV infection, and HCC family history.

Supplementary Table S5: Results of rs1776181 stratification analysis

Variables	Genotypes	Controls (n=1199)	Cases (n=1196)	OR (95%CI)[a]	*P*
Smoking					
No	CC	547	365	1	0.248
	CT/TT	461	384	0.834(0.613~1.135)	
Yes	CC	97	244	1	0.663
	CT/TT	94	203	1.109(0.696~1.769)	
Alcohol Consumption					
No	CC	47	41	1	0.607
	CT/TT	989	744	0.925(0.688~1.245)	
Yes	CC	7	23	1	0.54
	CT/TT	156	388	0.854(0.515~1.415)	
HBV Infection					
No	CC	48	13	1	0.607
	CT/TT	1053	182	1.088(0.566~1.578)	
Yes	CC	6	51	1	0.549
	CT/TT	92	950	0.629(0.139~2.858)	
HCC family history					
No	CC	54	59	1	0.493
	CT/TT	1135	1071	0.915(0.708~1.181)	
Yes	CC	1	5	1	0.494
	CT/TT	9	61	2.912(0.137~62.104)	

[a]: Adjusted by logistic regression for age, gender, nations, smoking, alcohol consumption, HBV infection, and HCC family history.
